# Pattern-based hybrid book recommendation system using semantic relationships

**DOI:** 10.1038/s41598-023-30987-0

**Published:** 2023-03-06

**Authors:** Fikadu Wayesa, Mesfin Leranso, Girma Asefa, Abduljebar Kedir

**Affiliations:** Information Technology, School of Computing and Informatics, College of Engineering and Technology, Wachemo University, Hosaena, Ethiopia

**Keywords:** Energy science and technology, Computational science, Computer science, Information technology

## Abstract

In the fields of machine learning and artificial intelligence, recommendation systems (RS) or recommended engines are commonly used. In today's world, recommendation systems based on user preferences assist consumers in making the best decisions without depleting their cognitive resources. They can be applied to a variety of things, including search engines, travel, music, movies, literature, news, gadgets, and dining. A lot of people utilize RS on social media sites like Facebook, Twitter, and LinkedIn, and it has proven beneficial in corporate settings like those at Amazon, Netflix, Pandora, and Yahoo. There have been numerous proposals for recommender system variations. However, certain techniques result in unfairly recommended things due to biased data because there are no established connections between the items and consumers. In order to solve the challenges mentioned above for new users, we propose in this work to employ Content-based Filtering (CBF) and Collaborative Filtering (CF) with semantic relationships to capture the relationships as knowledge-based book recommendations to readers in a digital library. When proposing things, patterns are more discriminative than single phrases. To capture the similarity of the books that the new user had retrieved, the patterns were grouped in a semantically equivalent manner using the Clustering method. The effectiveness of the suggested model is examined through a series of extensive tests employing Information Retrieval (IR) evaluation criteria. Recall Precision and F-Measure, two of the three widely used performance measuring metrics, were employed. The findings demonstrate that the suggested model performs noticeably better than cutting-edge models.

## Introduction

There is no shortage of content in the modern era. There are options for generating and gaining access to different types of data. People struggle to understand what to access for their requirements and areas of interest when there is a variety of content available. The largest issue arises when a person has too many options and needs to gather sufficient data to make an informed decision. It could be appropriate goods or services. There are many different ways someone's quest for a book to read could turn up, for instance, if they have no specific idea of what they desire. She or he can squander a lot of time exploring distinct websites in the hopes of succeeding. They might look for a recommendation from other people^1^.

Popular Web applications such as Amazon, Facebook, or Netflix use a recommendations approach to suggest new products/services to their users since navigation from page to page does not satisfy the user's need in a large amount of data. Predicting or assisting the users with their wishes about items (products) like books, electronics, and others is very important in E-commerce sites whether based on their recent browsing history or some hidden patterns as a limited context. Some systems, like Google Ad Sense, focus based on keywords than an estimation of the user’s taste based on her/his recent browsing history^2,3^. Recommender systems provide users with relevant items (top-k ranking list of “best” items) based on the user’s profile (information) they gathered about a specific user. Such types of Recommender Systems are called Personal recommendations. The context may rely on the user's current activity or her/his long-term interests. Again, the recommendation is done based on the information about the products like ratings and all details about the items.

The reviews and ratings are collected from users about the items through an implicit or explicit approach^4,5^.

- Explicit profiling is collected by asking each visitor to fill out the information about a specific item and how much they liked it (page, book, movie, news, CDs, hotels, products, services like transport) by providing a numerical rating.
- Implicit profiling is gathered when a user watches a movie or opens pages the system tracks the visitor’s behavior as interest. This technique is generally transparent to the user where browsing is tracked by recording specific users’ history (User identification) and behavior of user’s information^6^. Amazon logs each customer’s buying history and, based on that history, recommends specific purchases. There are the following approaches for Recommendation Systems^2,6^.

1. Collaborative Filtering: This approach builds a model from a user’s past behavior as well as similar decisions made by other users to predict items that the user may have an interest in.
2. Content-based Filtering: In the content-based filtering approach the characteristics of an item are analyzed to recommend items to the user.
3. Demographic Recommendation: Technique that uses only user's information to find a correlation between the users based on their demographic profile. Users with a similar demographic profile are recommended. The demographic technique suffers from a cold start problem for the new item, as the new item has not been preferred by any user of the same demographic profile.
4. Knowledge-Based Filtering: Items are recommended to a customer by using the knowledge of the item domain. It collects the customer’s preferences on a specific product and uses its knowledge to find the products according to the customer’s preferences.
5. Mobile Recommender System: Recommender approaches make use of internet accessing smartphones to offer personalized, context-sensitive recommendations. They always focused on spatial and temporal data^6,7^.
6. Hybrid The combination of the above or other approaches

Any user with a history or existing users might not be a problem to be recommended an item even if the recommendation does not fit the exact user's need. But a prediction for a new user which has no demographic input data faces problems. Another problem that leads to poor recommendation is an item that has no rating value or a new item. The aim of this paper is interested in developing a hybrid book recommendation system that applies a pattern or data mining rule to find the correlation between books that are to be recommended for the new user who has no previous history. The pattern in the data mining could facilitate the new users as well as for new items which have less or no rating value when searching for books, and give better-recommended results. In addition to pattern features grouping them to identify the related items, as well as similar user interest, is very important. The main research contributions are summarized as follows:

- We propose to exploit Content-based Filtering (CBF) and Collaborative Filtering (CF) with semantic relationships to capture the relationships as knowledge-based to recommend books to readers in a digital library to address the problems stated above for new users.
- We model the patterns which are grouped to a semantically equivalent approach to capture the similarity of retrieved books for the new user using the Clustering method.
- We use an extensive experiment and the three popular performance measurement metrics; Recall Precision and F-Measure to evaluate the effectiveness of the proposed models by using Information Retrieval (IR) evaluation metrics.
- The results show that the proposed model significantly outperforms state-of-the-art models.

The rest of this paper is organized as follows. “Related works” explains the existing research works. “Methods” formulates the problem in this paper. Then, we propose an algorithm to solve the formulated problem. The simulation results are illustrated in “Experimentation result, discussion and evaluation”. Finally, we conclude this paper in “Experimentation result, discussion and evaluation”.

## Related works

Researchers have been working to boost the performance of recommender systems using an integration of more than one technique. Various hybrid approaches have shown good results. In^8,9^, the authors suggested a technique that introduces the contents of products into the product-based collaborative filtering system to improve the performance of a prediction algorithm. It is called the product-based clustering hybrid approach. In this approach, they first applied the clustering algorithm to group the products. The main purpose was to group the products into various sets and provide content-based information to determine similarities. Each product has its attributes, such as the movie product, which may have an actor, actress, director, etc. Thus, they grouped the items based on those attributes.

In^10,11^, a hybrid recommender system that integrates collaborative and content-based approaches has been adopted. Firstly, the content-based filtering algorithm is applied to find customers, who share similar interests. Secondly, a collaborative algorithm is applied to make predictions. It integrates the product information and product ratings to calculate the product-product similarity, called product-the based clustering method. It also integrates a customer’s information and a customer’s ratings to calculate the customer–customer similarity, called the customer-based clustering method.

In^10,12^, authors suggested a content-based predictor to improve already-existing user data, and then used collaborative filtering to produce tailored suggestions. In order to handle a vector of bags of words, they constructed a bag-of-words naive Bayesian text classifier, where each bag-of-words corresponds to a specific aspect of a film, such an actor or a director. Additionally, they learned a user's profile from a collection of rated movies using the classifier. The rating of unrated movies is then predicted using the learned profile. The neighborhood is built using the Pearson correlation algorithm and user-based collaborative filtering.

In^11,13–15^, the writers suggested more A hybrid recommender system for tourism can give useful information on tourist attractions based on a user's profile, location, schedule, and the amount of time they have to visit their preferred locations. Mobile technology has developed to provide useful communication and computation functions. The authors proposed a platform that enables users to decide based on their location, schedule, context, and mobility requirements as a result. The smartphone and a server are the two parts of the suggested system. With a smartphone, the user is connected to the system and has access to all functions at any time. A server offers the user a number of functionalities including presentation, recommendation, punishment, socialization, and advertising.

In^8,16^, to increase prediction accuracy, provide better coverage, and address the cold start issue, the authors suggested a hybrid strategy that combines content-based, collaborative, and demographic filtering techniques. By classifying the consumers into several categories using the closest neighbor technique, the demographic characteristics of the customers (e.g., gender, race, age, employment status, occupation, etc.) are used to solve the cold start problem. To locate the closest neighbors, they applied the KNN algorithm^5,14^. The authors assessed and contrasted their hybrid algorithm with other approaches. According to their findings, their strategy outperforms the competition for both the cold start issue and for all consumers.

Each category contains readers sharing similar demographic characteristics. The combination of demographic characteristics and content-based approaches allows for solving the problem of new items that are added to the system. In the related works, there is no approach try to present the problem of the items to a user in a specific order across the most popular. This ignores the rare rated items (newly added items) or not popular ones.

On the other hand, some other approaches consider user profiles in the recommendation process since the profiles represent the users' information needs to identify the needs of an individual user. The accuracy of each user profile affects the performance of the entire recommender system. If a new user has no history, the recommendation might not be effective or difficult.

So this paper suggested enhancing the quality of recommended items and the problem of cold start as well as to improve the scarcity problem, integrating knowledge base like using extraction of the relation between entities better-using clustering method. After the clustering has been done based on the probability approach the books to be recommended were filtered and the similarity has been calculated. The focus of this is to provide justifications for recommended books as this plays a crucial role in obtaining the satisfaction and trust of readers. It has been shown that a reader’s trust is positively associated with a reader’s intentions to read a book.

## Methods

**Figure 1:**
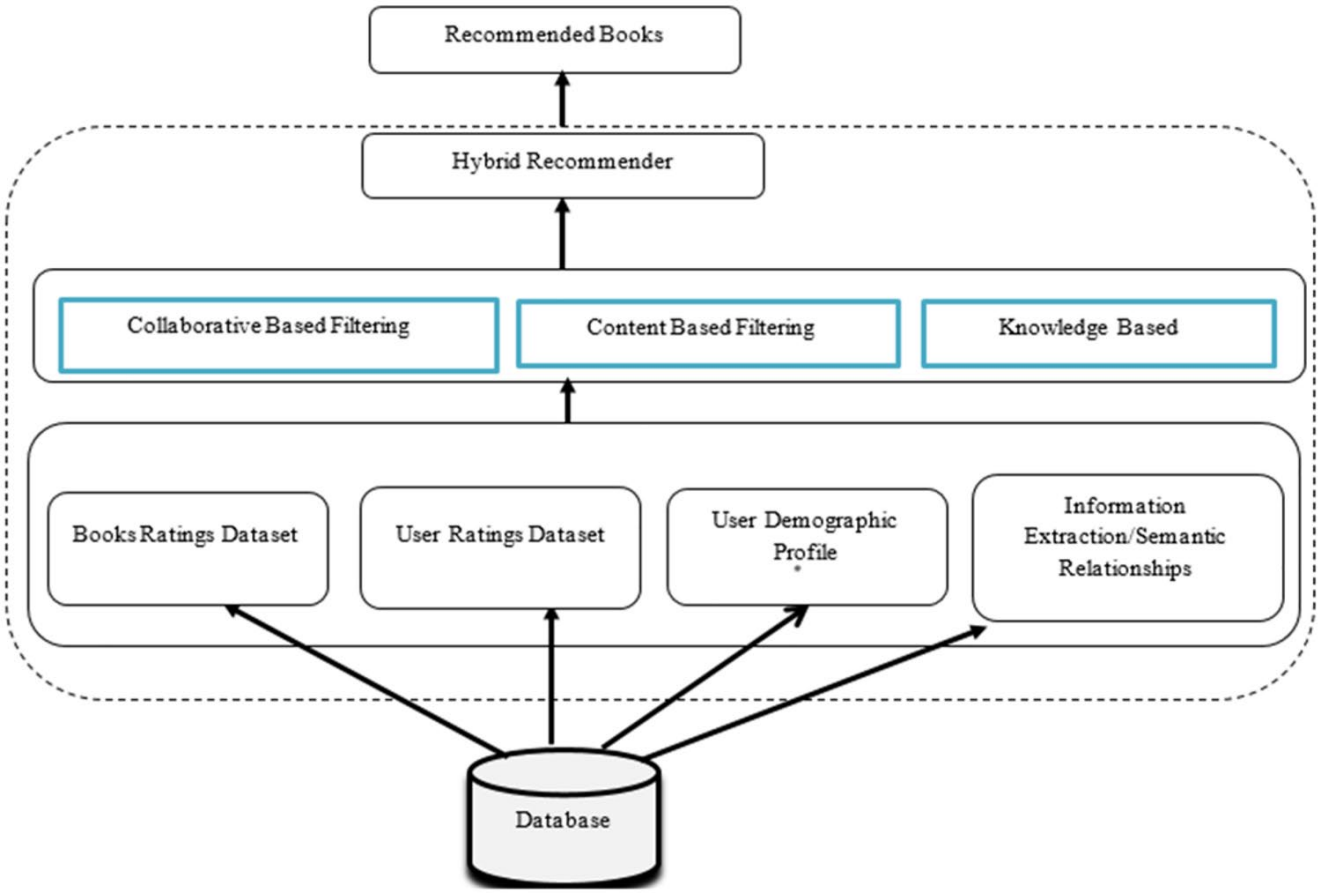
The proposed framework.

Figure 1 depicts the overall procedures considered in this work. Let we see each components each one by one as follows.

### Components of the Proposed Model

1. Clustering Data: We grouped user demographic data, books, and rating data of books by users because the number of readers and books is large and it is difficult to search and compare the similarity of each user with all existing users and we might not get the required user need. So, we applied the clustering algorithm to group all reader data, books rating, and book information to get the similarity of the data in our database.
2. Clustering Users Data-set: We clustered existing user data-set by grouping users based on the attributes we selected from user demographic information to cluster them. The existing users are clustered to their appropriate group based on their similarity according to their category. In our case department is used. We clustered users based on their department. We select this attribute since we have the assumption that readers with a similar department have a similar interest in books because the reader wants to know the information on their department every time.

This clustering is important and we use it when we search for similar users for the newly registered users for reducing search complexity to find the specific user groups needed to be recommended. Since we are recommending new users, it is better to know the user’s group by identifying the registered information about users. We check always similar groups for the registered new users based on the department on the online process. When a new user registered the process of searching for the appropriate cluster for the user will continue and if the cluster is found the recommendation is done. But if the new user couldn’t get the exact groups, the new groups for the users are created in our system. In addition, the recommendation provided for this new user is the popular books filtered. The algorithm for user clustering is as follows.

In the scenario of finding a relationship between the books, we take into consideration not only the magnitude of each word count of each book but also the angle between two vectors can be calculated as:

1 $${\text{cos}}\theta = \frac{{\overset{\lower0.5em\hbox{$\smash{\scriptscriptstyle\smile}$}}{a} .\overset{\lower0.5em\hbox{$\smash{\scriptscriptstyle\smile}$}}{b} }}{AB}$$

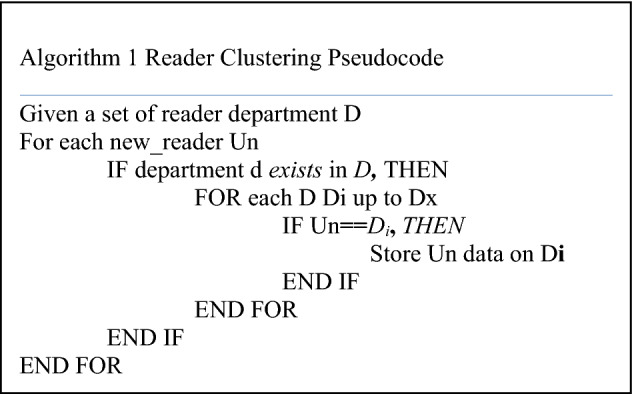

- Clustering Books Data: According to Algorithm 1, the data set identifies books read and rated by users which are stored in the table with different features of books like ISBN, categories, Title, Author, Year, publisher, and others. So, to access the books currently existing in the system we need to have a better way to process and retrieve from the dataset. Since they are many books and take too much time and memory for processing and retrieving them. The better way to overcome this problem is by using clustering methods. We clustered this book's dataset based on the category. This means the books with similar categories are clustered under the same cluster. For example, the books in the department of Information Technology should be considered under the Information Technology cluster. Generally, the clustering reduces the time used to process and the memory used while retrieving the required books and unnecessary data. So, this method overcomes the salable problem one of the common problems in the books recommendation system.
- Clustering Rating Data: This dataset contains the books rated by users with the user ID and Books IBN (ID). In our model, the new user registered for the system looks for books, and the system provides books requested by a user based on their rate values. The processing of this huge data takes many time and memory which follows the scalability problem. We apply the clustering method to group the rating dataset. As it is mentioned this rating dataset has different features and we used these features for clustering. The clustering for this dataset is done based on the rate value of books given by the users who read and rated the books. After we clustered them we have 4 groups of books. Those are above-average books rating data which contains books rated by the user with the 7 to 10 rating value, average rating data with 6.5 rating value, medium rating data with 3 to 5 value and below rating data with 1 and 2 rating values, and visited rating data with no rating value but visited by the readers. We also used reader behavior information that is gathered from reading history, and readers' ratings on books. Reader behavior information is gathered by giving ratings to the books on a scale of 1 to 10 is used by the readers, where 1 indicates a less favorite and 10 indicates a top favorite book. The books that have no rating by the reader are indicated by 0. This rating information creates contextual information for the system and is shown as follows:
  $${\text{R}}:{\text{Readers}} * {\text{Book}} * {\text{Context}} \Rightarrow {\text{rating}}$$
- Clustering New Users: The registered user groups should be identified according to user similarity and the data are registered to the appropriate cluster or the similar groups. This identification of new users is done based on demographic attributes. The system adds the user demographic data into his/her respective group. The users clustered under similar users based on their department. Filtering this information will reduce the number of users by considering that users with similar age groups will have a more similar interest in the books to be read.

We used Information Extraction (IE) to capture the relationship between books and authors to suggest a given book as a sample is indicated in the algorithm 2. We generated the triples to represent a couple of books and a relation between them. For example, (Michael T. Goodrich, write, Data structure and Algorithms) is a triple in which ‘Michael and ‘Data structure and Algorithms’ are the related entities, and the relation between them is ‘write’. We used a Rule-based Approach by defining a set of rules for the syntax and grammatical properties to extract information from books and used it as input for a recommendation.
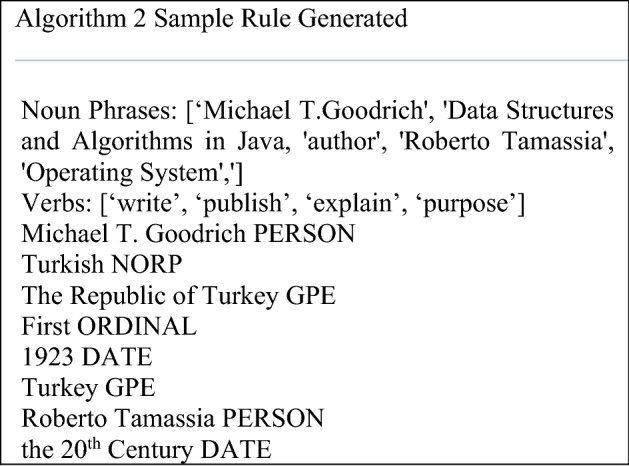


According to the Algorithm 2, before any book is recommended to the user’s semantic relations were generated from the books information using a pattern relationships. Then, based on the constructed relationships, the most relevant books are recommended.

- Retrieving Most Highly Rated Books: The rated books by filtered users should be fetched and the process of priority consideration is applied to them before predicting the newly registered user. The user is provided a list containing 10 highly rated books to ensure there is a representation of highly rated books.

Our approach used the following methods to predict recommendations. These are relevance (most highly rated), clustering, and popularity (most highly read). The recommendation is done in both content-based and collaborative filtering approaches.

1. Filtering Books by content-based approach: The book categories are used to cluster the books rated by users and these books are rated by different users. So the books rated by many users from one category should be recommended for the new users similar in the cluster in addition to recommended based on the collaborative filtering.
2. Filtering Books by collaborative filtering approach Books rated by the user under one group according to their demographic similarity are filtered. Since the rated books are clustered into four different groups, the system should check from all groups and follow the priority to return the books. The books with the highest rating value should get priority if they fulfill the number of books to be generated for the readers. If there is a concerning book for the group, nothing is recommended. Predicting of rating value for each book to be recommended for this new user will be done by the given rating value for each book rated in the new user cluster since the new user has no rating values for any of the books. Predicting his/her rating based on the groups of the user is the task of this work to predict the rating value of this user. To predict the rating value, the system will calculate the weight of high-rated books by the user in similar groups for the new users. After the weight of the rating is calculated, the books with that weight value will be recommended to the users.
3. Filtering popular Books: The popular books assumption in this work with the highest rating value and rated by many users is recommended. We check their frequency or the number of occurrences since the books frequently occurred the books are rated by many users and it is popular with many readers. Our model takes input upon which to base the recommendations. The input used in our model includes a reader's interest profile, rating data, and book information. Accurate readers’ information has a crucial role in integrating different recommendation techniques. Reader’s profile describes the reader's description information such as a department. We gathered this information during reader registration. Finally, we retrieve these books by checking their rating value and retrieving all with the highest rating value.
4. Generating Top Recommended Books: The top N articles recommendation is done by the ranking algorithm we developed for the books recommendation system. Since the information our work recommend is the books we need to consider the time the book's articles were published and the popularity of the books by readers. The books to be recommended are retrieved through both the two approaches content-based and collaborative filtering and the books are generated based on popularity.

Since each of the approaches we have used has its ranking methods for the books to be generated and the results obtained are generated by the ranking algorithm each of the approaches used. And the results obtained in individual approaches are combined. Finally, we generate the books to be recommended by the time they were published. This means the recent books are displayed at the top and the next is also continues in this order.

## Experimentation result, discussion and evaluation

This part examines the application of the experimental findings to the recommendation problems as well as the evaluation of the proposed recommendation methodologies, datasets, and evaluation criteria.

### Dataset

This research uses the good books dataset^17^, Harper and Konstan, 2015 which is a commonly used dataset in the domain of recommender systems. It contains the results of real users’ interactions with the recommender system. It can recommend books using the user profile. The availability of the content descriptions helps in finding similar books to the one selected. It is designed to offer user-item matrices to be used to develop recommendation algorithms. This dataset was chosen to evaluate the developed algorithms in this research, and show their effectiveness and novelty compared with the algorithm. This dataset contains the text file of books with the book's ISBN, title, author, publisher, categories, published year, and URL of books. We have changed the text data of books from the source into a table in our database according to their attributes appropriately.

The dataset consists of 326,376 ratings using numerical values ranging from 1 to 10, from 278,850 users based on 271,379 books. Users who had made fewer than 20 ratings were removed from the analysis. In addition, this dataset contains demographic information for each user including details of their department, year, and semester. Users who did not complete these details were removed from the analysis.

We have demographic information of users who rated or read the books with their department, semester, year, and user ID. The demographic information of users is stored in one table. The rating value given for each book by the active users is also another data we used in this study and it is stored on another table.
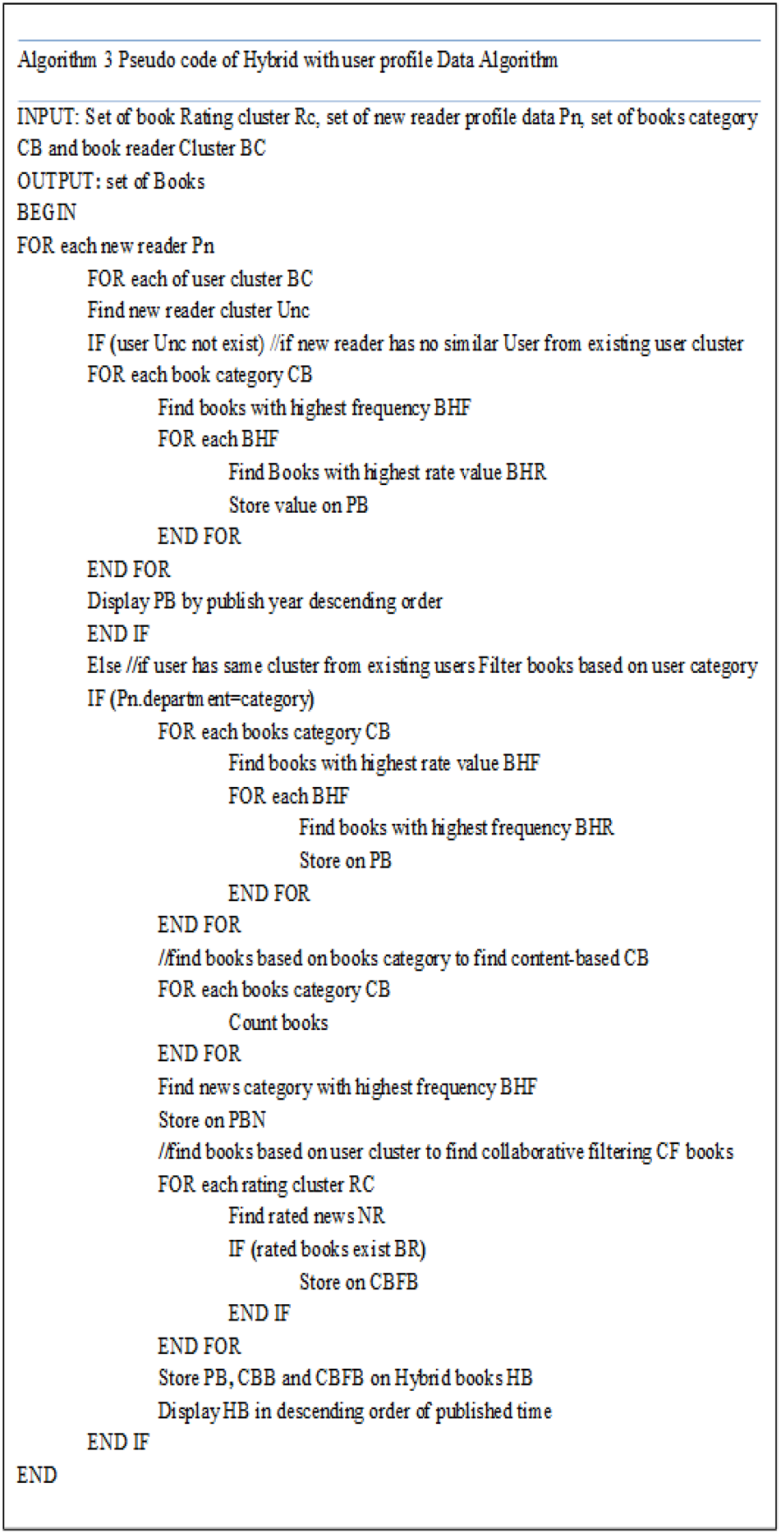


As we can see from Table 1, the user dataset contains two attributes; those are User ID, and department or category. The predefined features were extracted for the books to gain a rating matrix of items by a set of users, user’s description (sex, age, location, profession of the user), and Items' description (genre, author, title, date, price of the item). Recommender systems researchers have applied different measures to evaluate recommendation algorithms in terms of accuracy and quality. This insight is useful for evaluating the quality of a system and its ability to forecast the rating for a particular item. To evaluate the performance, we depend on the objectives of our study and we selected the related metrics to our objectives. Since the main objective of our study is to recommend more related or interested books to users we should have to evaluate the relatedness of the books to users. So, the popular and the most used metrics for any information retrieval to measure the relatedness or interests are precision, recall, and F1-score.

**Table 1:** Books dataset information.

UserID	ISBN	Book rating	Department
276726	0155061224	5	HO
276729	052165615X	3	Med
276729	0521795028	6	Med
276744	038550120X	7	CS
276747	0060517794	9	CS
276747	0671537458	9	CS
276747	0679776818	8	CS
276747	0943066433	7	Med
276747	1885408226	7	Med
276748	0747558167	6	Bio
276751	3596218098	8	IT

### Implementation tools

Both the backend and the frontend of the prototype have been implemented using several tools. Our study's implementation tools included the Java programming language, NetBeans 8.0.1 tools for building the model on the front end, and MySQL 5.1 for storing and processing the dataset on the back end through a connection to NetBeans. The prototype of our approach is implemented using the NetBeans tool, which is also used to create new user accounts and deliver output to users.

### Evaluation metrics

Table 2 shows how to evaluate the performance of our model in terms of Top-N recommendations, the classification accuracy metrics (i.e. Precision at N, Recall at N, and F1 measure) were chosen for the accuracy performance evaluation of the recommender against the users in the test set.

1. Precision: This result will be found by calculating using Eq. (2),^1^.
  2 $$P=\frac{TF}{TF+FP},$$

**Table 2:** Evaluation metrics table^11^.

	Recommended	Not recommended
Relevant books	TP (true-positive)	FN (false-negative)
Irrelevant books	FP (false-positive)	TN (true-negative)

Where P states the Precision, FP states that relevant for all books recommended.

2. Recall: In our case, it measures the good recommended out of all good recommended items as described in Eq. (3),^1^.
  3 $$R=\frac{TF}{TF+TP},$$

Where *R* states Recall, *FN* states that relevant for all books recommended.

3. F1-Score: F1-score is the harmonic mean value of both precision and recall results as its formula shown in Eq. (4).
  4 $$F1-Score=\frac{2TF}{2TF+TF+FP}.$$

### Evaluation result

The model we used in this study uses the data mentioned above to provide the output from the screenshot for the users, and we evaluate the performance of the final output result provided for users by the metrics we have used to measure the performance of the system. We evaluated the performance of our works in two ways. One is the experimentation of our work for comparing each user with each other and the other is the experimentation of the system for comparing the performance of the users with clusters that consist of many users. This work help in recommending the books to the users based on discovering the relationships among books and the users that allows users to combine their descriptive static profile with dynamic books behavior.

1. Experimentation for individual user similarity: For the individual users we evaluated by taking 20 users from 400 active users. We remove the books rated by these users and register each of these 20 users as a new user and we run our proposed model to recommend the books for the users and we compare the previous books rated by the user with these actual recommendations. Then, we calculate the precision, recall, and F1-score values as the formula we discussed in the previous section. According to this experimentation, the results of the works are explained in the Table 3 table format.

**Table 3:** Experimentation based on user similarity.

User ID	Precision	Recall	F1-Score
93589	0.740741	0.4578	0.5992705
132852	0.85121	0.5645	0.707855
255542	0.88451	0.6568	0.770655
19498	0.65985	0.3351	0.497475
44035	0.55123	0.2354	0.393315
Average	0.6384141	0.404274	0.521344

Below is a table positioned for experimentation based on user similarity using a sample:

From the evaluation results, we have the accuracy of the book for each user with the values of 63*.*84% of average precision, 40*.*42% of average recall 52*.*1% of the average F1 score values as shown in Table 3.

**Figure 2:**
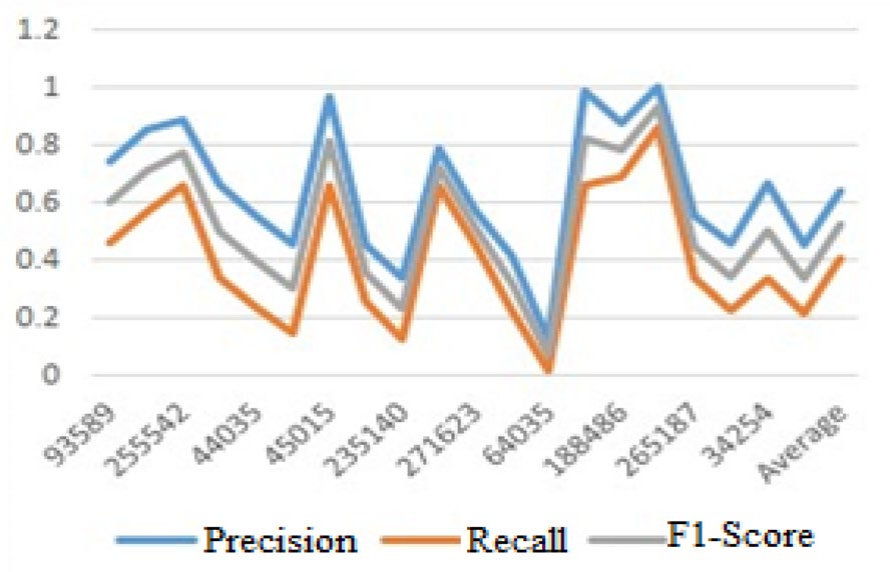
Experimentation value based on individual user similarity.

The above mentioned result can be represented graphically as shown in Figure 2 to show the sample performance results.

2. Experimentation by user cluster-based similarity: The other way we evaluated our performance is based on the user cluster. We took 4 user clusters out of the user clusters we have in our dataset. Then, we recommend some new users similar to the cluster selected and we compare the accuracy performance by comparing the actual recommendation with the recommended for that cluster. The results of the work are explained is shown in Table 4.

Based on this experiment we have the accuracy values that perform the average precision of 0.76416, average recall of 0.37429, and average F1-score of 0.56923, and the average precision of this experiment is shown in Table 4.

**Table 4:** Category based evaluation.

Category number	Precision	Recall	F1-Score
2	0.7745	0.36213	0.5992705
4	0.58652	0.13541	0.707855
6	0.8845	0.6568	0.770655
8	0.811124	0.3351	0.497475
Average	0.76416	0.37429	0.56923

### Discussion and conclusion

1. Discussion: This work employs Content-based Filtering and Collaborative Filtering with semantic relations to capture the relationships as knowledge-based book recommendations for the biased data of new users. We conducted two ways of experimentation mechanisms as experimentation based on individual user and experimentation as clustered user similarity. From the two experimentation results we gained quality book recommendation when compared to other previously work done.

The result of our experiment in this work contains two experimentation ways and each has different values as discussed in the previous section. Finally, we analyzed that the recommendation performance is different as obtained in two ways of our evaluation methods. The recommendation accuracy result is different according to the similarity of the users with clustering-based and individual user similarity. According to the two experiments results, we analyzed that the more accurate recommendation is done for the users in the clustering which performs the precision of 76*.*4% Recall of 37*.*4% and F1-score of 56*.*9% rather than individual user recommendation.

This is done because the cluster-based recommendation contains more related books regarding the users in that cluster than the books recommended for individual user similarity. According to this value, the Recall in the individual performs less performance than in cluster-based. The reason behind this result is the finding of good recommendation result numbers from many users in the cluster. So, if the good books recommended are many then, the Recall value will become less.

From the Table 5 above, it is evident that findings of the proposed models makes the best prediction with the highest accuracy score when compared to the other models.

**Table 5:** Result comparison of different algorithms with the proposed model.

Approach	Precision	Recall	F1 Score
Hybrid model (proposed model)	0.638	0.404	0.521
Semantic based STRuFSP^18^	0.563	0.479	0.484
Probase-LDA	0.413	0.327	0.429
CLDA^19^	0.38	0.37	0.401
Pattern based word embedding (proposed model)	0.764	0.374	0.569
Pattern based TNG	0.446	0.386	0.374
N-Gram	0.401	0.386	0.361
Frequent closed patterns-FCP	0.428	0.385	0.362

The Hybrid Model and Pattern based Word Embedding were the proposed models that was clearly the most accurately identified result with the overall accuracy of 0.521 and 0.569 F1-Score respectively. Hence, it is reasonable to assume that the extraction of semantic relationship of the books as described previously, the relatively positive overall evaluations model.

**Figure 3:**
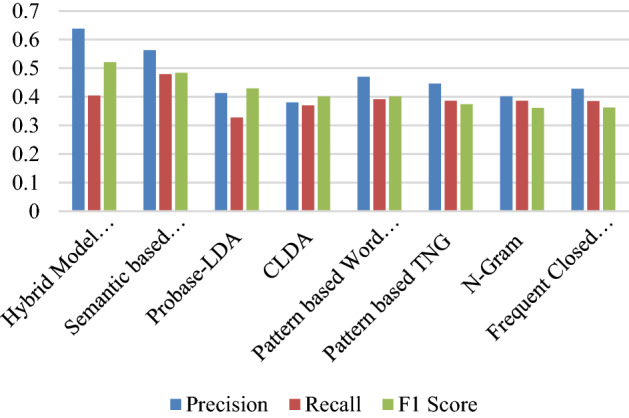
Result comparison of the proposed model with other models.

From the Fig. 3, our model performance evaluation was conducted a comparison with other models and it shows our proposed model achieved the best result among the models.

2. Conclusion: Educational domain is based on a heterogeneous collection of information and services. These services are student information services and digital library services. The main objective of this study was to design a recommendation system for a digital library. There are many challenges in the recommendation system as we discussed in the related work. In this study, New User profile data with a Hybrid book Recommendation system was proposed. This hybrid recommender scheme combines a content-based and collaborative approach with user profile information with the help of pattern relationship between the users. The content-based component uses the book features to get knowledge about the content type of the books to select the recommendation for a similar user in the same cluster rate for mostly rated categories of books.

## Supplementary Information

Supplementary Information 1.

Supplementary Information 2.

Supplementary Information 3.

Supplementary Information 4.

## Data Availability

All data generated or analyzed during this study are included in this published article [and its supplementary information files.
